# Impact of Water and UV Irradiation on Nonwoven Polylactide/Natural Rubber Fiber

**DOI:** 10.3390/polym13030461

**Published:** 2021-01-31

**Authors:** Yulia Tertyshnaya, Maria Podzorova, Maksim Moskovskiy

**Affiliations:** 1 Department of Biological and Chemical Physics of Polymers, Emanuel Institute of Biochemical Physics, Russian Academy of Sciences, 4 Kosygina Str., 119334 Moscow, Russia; yersinia@bk.ru; 2 Federal Research Agro-Engineering Center VIM, 1st Institutskiy Proezd, 5, 109428 Moscow, Russia; maxmoskovsky74@yandex.ru

**Keywords:** polylactide, non-woven fiber, natural rubber, hydrolytic degradation, UV-irradiation, electrospinning

## Abstract

A nonwoven fiber made of polylactide/natural rubber with a rubber content from 0 to 15 wt.% was obtained by electrospinning from a solution. The water sorption test showed that the addition of natural rubber into the polylactide matrix did not significantly affect the degree of water absorption of the fibers, which was in the range of 48.9–50.6%. The melt flow rate only increased by 0.5 g/10 min at a content of 15 wt.% natural rubber. The thermal characteristics after 120 days of degradation in distilled water and UV irradiation (50 h) at a wavelength of 365 nm were determined using differential scanning calorimetry. Changes in the values of the phase transition temperatures and the degree of crystallinity were determined. It was determined that the fiber samples from all compositions retained the propensity for photo- and hydrolytic degradation.

## 1. Introduction

The modern agricultural sector is in search of innovative materials and technologies to improve the process of crop cultivation, increase productivity, and develop breeding. The use of biodegradable fibrous polymer materials may be suitable for producing seed mats, covering material, air-permeable packaging, etc. [1,2].

Polylactide (PLA) is polyester obtained from renewable raw materials. Lactic acid is released during the fermentation of waste vegetable raw materials: beets, corn, cereals. At the next stage, polylactide is obtained from lactic acid by polymerization [3,4,5]. Crystalline PLA has properties similar to those of known polymers, such as polypropylene and polyethylene, but they have advantages: biocompatibility and biodegradability [6,7,8,9]. Under the influence of environmental agents, PLA decomposes to carbon dioxide and water.

Interest in PLA is only growing, and research on the study of film and fiber materials from PLA is currently being actively conducted [10,11,12]. Yang et al. [11] studied the morphology of the PLA fibers and their potential in neural tissue engineering. Nicosia et al. reported about the manufacturing of a PLA/PHB fibrous mat with antimicrobial properties for aerosol particle filtration [12]. Ultrathin and nanoscale fiber matrices are of great interest for medicine and the agricultural sector for creating functional materials with biocompatibility, bioresorption, effectiveness and safety properties for living organisms and the environment. The functionality of such materials is due to the high surface-to-volume ratio of an individual fiber. The possibility of modifying its surface is regulated by physical and mechanical characteristics and features of diffusion transport in combination with the use of natural biopolymers. Currently, ultrathin and nonwoven fibers and products from natural biopolymers are actively used in biomedicine, cell engineering, separation and filtration processes, sensory diagnostics, and in a few other areas of science and technology [13,14,15].

One of the most promising methods of producing nano- and micrometer-range fibers is electrospinning, which makes it possible to produce fibers and nonwoven materials from biopolymers. Electrospinning is a multiparametric technological process for producing micro- and nanofibers with a diameter in the range of 10 nm–40 microns. The electrospinning process is subject to a combination of mechanical and electrostatic forces operative on a polymer solution or melt-oriented in an electric field. A number of parameters, such as the voltage at electrodes, the distance between electrodes, and the conductivity, density and viscosity of the molding polymer system, affect the geometry, surface properties, porosity, supramolecular and molecular structure, and fiber functionality [16,17].

The blending of PLA with other polymers is less expensive and more practical. Rubber has been used as a second-phase polymer to toughen brittle thermoplastics. Natural rubber is a high-quality polymer derived from commercial latex, which is obtained from the milky juice of a rubber-bearing plant—Hevea. Natural rubber has been shown to be biodegradable by various mold fungi and a wide range of bacteria [18,19]. The repeat unit is 1,4-cis isoprene, and the polymer is widely used to create composite materials [20,21]. Pongtanayut et al. [21] reported that the ductility of PLA was significantly improved by blending it with NR. The amount of NR at 10% weight seems to give an optimum property. It has been reported that NR may be distributed in the form of flexible nanoscale or submicron formations in a polyolefin matrix to produce composites with a greater elongation than that of the polyolefin [22]. A similar effect was observed when NR was mixed with polylactide to obtain film samples [23]. Xu et al. [23] also found that a reduced PLA phase in the blend and the poor interfacial adhesion between PLA and NR could be the reasons for a decreased tensile strength and hardness. The production and study of PLA/NR fibers has not been reported.

A new composite nonwoven fiber material made from polylactide and natural rubber (NR) has been generated [24]. Nonwoven PLA/NR fiber has an increased elasticity compared to that of pure PLA, while remaining eco-friendly and biodegradable. The current study aims to investigate the influence of environmental factors such as water and UV irradiation on the degradation of PLA/NR fiber.

## 2. Materials and Methods 

Polylactide 4032D with a molecular weight of 1.7 × 10^5^ g/mol and ρ = 1.24 g/cm^3^ produced by Nature works (Minnetonka, MN, USA) as well as natural rubber SVR 3 L from Vietnam Rubber Group (Ho Chi Minh City, Vietnam) were used. The natural rubber consisted of poly(cis-1,4-isoprene), with minor impurities from other compounds and water (Table 1) [25].

The polymer solutions for electrospinning were prepared by dissolving PLA and PLA/NR in the right ratio in chloroform. The mixtures were heated at 60 °C for about 4–5 min. The polymer solution was placed in a syringe with a needle inner diameter of 0.7 mm, set up vertically. The electrospinning experiments were performed at room temperature (20 ± 2 °C). The consumption of the solution was (9–11) × 10^5^ g/s. The content of natural rubber in the mixture was 0, 5, 10, and 15 wt.%

The kinetics of distilled water absorption by the samples was studied for 240 h after the materials reached equilibrium water absorption (GOST 4650-2014 (ISO 62:2008)). Square samples with a side of 30 mm were used for testing. The test was performed for five samples of each composition. Before the test, the samples were dried at (40 ± 2) °C for 24 h and then cooled in a desiccator over a desiccant at (22 ± 2) °C and weighed no more than 5 min after removal from the desiccator. Furthermore, the samples were placed in a vessel with distilled water taken in an amount of at least 8 cm^3^ per 1 cm^2^ of the sample surface. The test samples were not allowed to be in contact with each other or the walls of the vessel, and were completely covered with water. The liquid was agitated by rotating the vessel at least once a day.

When determining the maximum water absorption, the equilibrium was considered reached if the difference between the sample mass, determined at an interval of 24 h, did not exceed 0.1%.

After an equilibrium amount of water in the sample was established, it was removed from the water, dried with a filter paper, and weighed using an electronic balance no more than 1 min later.

The degree of water absorption was calculated as (1):

(1) $$W=\frac{\left(m_{2}-m_{1}\right)}{m_{1}}\times 100\%$$

where *m*_1_—initial mass of the sample, and *m*_2_—mass of the sample after water influence.

The resistance of the samples to photodegradation was studied using a UV radiation source VL-6.LC from the company Viber Lourmat (France). The wavelength was 365 nm. The distance from the lamp to the samples was 15 cm.

The melt flow rate (MFR) was determined in accordance with GOST 11645-73 (ISO 1133-76) on the device IIRT-5 (Russia), which was based on the principle of a capillary viscometer.

The thermophysical characteristics of the samples were determined by differential scanning calorimetry (DSC) using a DSC 204 F1 device (Netzsch, Selb, Germany). The scanning speed was 8 deg/min, the weight was varied in the range of 5–5.5 mg, and the calibration was performed for indium with a melting point T_m_ = 156.6 °C. The accuracy of the measurement was T = 0.1 °C. The temperatures of melting and crystallization (T_m_ and T_c_) were determined by an endothermic maximum of the melting peak and an exothermic maximum of the crystallization peak on DSC thermograms, respectively

The value of the degree of crystallinity *χ_cr_* was calculated by Formula (2):

(2) $$\chi_{cr}=\frac{\Delta H_{m}}{\Delta H_{m}^{*}}\times 100\%$$

where $\Delta H_{m}^{*}$—enthalpy of melting of the ideal crystal, which was 93 J/g for PLA [3].

The thermogravimetric analysis (TGA) of the samples was carried out in argon on a TG 209 F1 thermoanalytical balance (Netzsch, Germany) at a heating rate of 20 K/min.

The experimental results were calculated as the arithmetic mean and its standard error. The calculations were performed using Statistica 8.0 software (Dell Software Inc., Round Rock, TX, USA) and Microsoft Excel 2007.

## 3. Results

**Figure 1:**
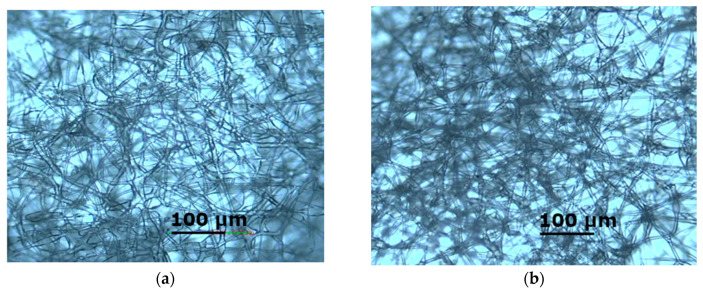
Microphotos of nonwoven fiber: (**a**) 100% PLA and (**b**) 95% PLA/5% NR. Magnification of 100 times.

The PLA/NR agrofiber of various compositions was obtained from the solution by electrospinning. PLA/NR nonwoven fiber (Figure 1) can be used in agricultural applications as seed mats or covering material, for example to protect the breeding seeds of experimental crops from birds and adverse weather. 

**Figure 2:**
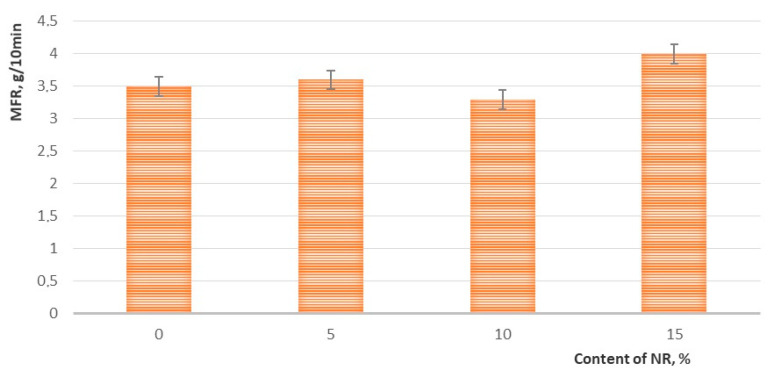
Melt flow rate of samples of nonwoven PLA/NR fiber containing 0, 5, 10, and 15 wt.% of NR.

When using any polymer materials, both film and fiber, it is important to know the rheological properties, so for all samples of nonwoven PLA/NR fiber, the melt flow rate (MFR) was determined (Figure 2).

As the experiment showed, the addition of natural rubber did not significantly affect the MFR of PLA/NR samples; only when the NR content was 15 wt.% did the MFR increase by 0.5 g/10 min, which was a completely acceptable change in MFR for polymer compositions.

**Figure 3:**
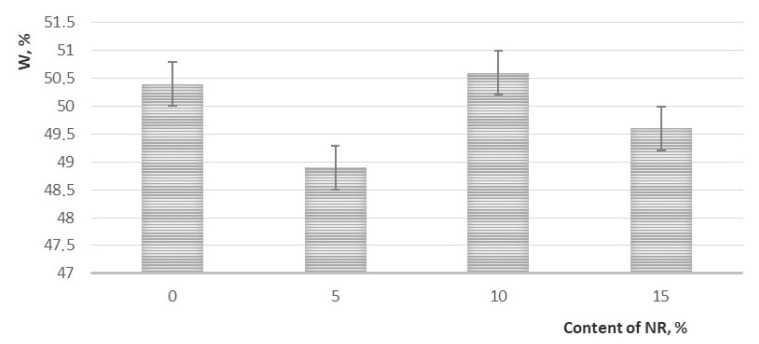
Degree of water absorption (W, %) of samples of nonwoven PLA/NR fiber containing 0, 5, 10, and 15 wt.% of NR.

When using fiber in open ground conditions, the main environmental agents affecting PLA/NR fiber will be water and ultraviolet irradiation. The water absorption test allows one to determine the equilibrium amount of water absorbed by the material under certain conditions. The water adsorption by polymers depends on many components. For example, the degree of crystallinity and the temperature play an important role. The degree of water absorption is very dependent on the type of material. It is obvious that due to the porous structure, the fiber absorbs more water than the film. In this case, it is the type of material that plays the main role, and not the hydrophilicity or hydrophobicity of the polymers in the composite. The experiment showed that the addition of natural rubber had almost no effect on the degree of water absorption *W* (Figure 3).

**Scheme 1:**
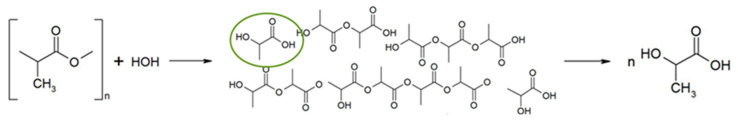
PLA hydrolysis.

Water is one of the most aggressive environmental factors. A large number of investigations have been performed on the hydrolytic degradation behavior of PLA films in aqueous medium [26,27,28,29,30]. As for the long-term exposure to the aqua environment, many studies have shown the hydrolytic degradation of polylactide according to the Scheme 1 [30,31]:

Hydrolysis of PLA occurs by the addition–elimination of a complex carbonyl induced by water, and then water-soluble oligomers and monomers are produced. In the process of hydrolytic degradation, the samples turn white. Such an effect was also observed for PLA/NR fibers.

**Figure 4:**
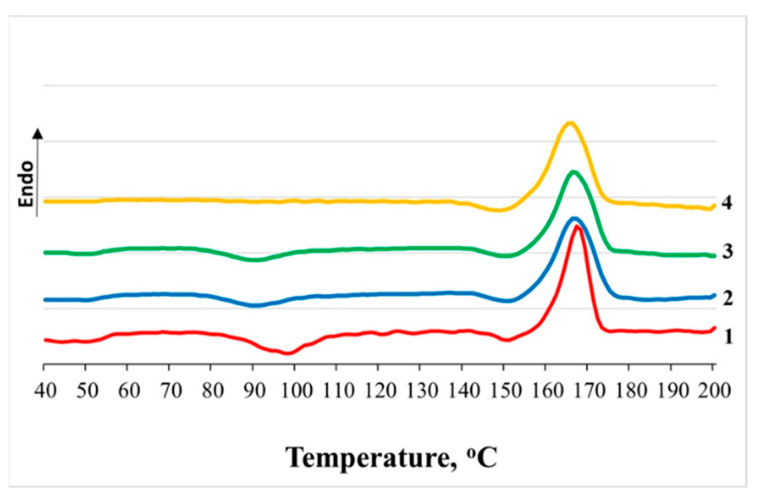
Melting thermograms of nonwoven PLA/NR fibers with different NR contents: (1) 0, (2) 5, (3) 10, and (4) 15 wt.%. 180 days of water degradation. DSC method.

When studying the effect of water on nonwoven fiber PLA/NR during 180 days, it was determined that after hydrolytic degradation on the melting thermograms, the melting temperature (T_m_) of PLA/NR samples changed insignificantly and the glass transition peak of PLA almost disappeared (Figure 4). Such a behavior of T_g_ is attributable to a decrease in the molecular weight [26]. 

An increase in the PLA degree of crystallinity is a consequence of the disintegration of the amorphous phase and a possible decrease in the molecular weight (Table 2). The remaining PLA chains then have a higher mobility, and they can reorganize themselves more easily, which leads to an increase in the degree of crystallinity [27,29]. 

**Figure 5:**
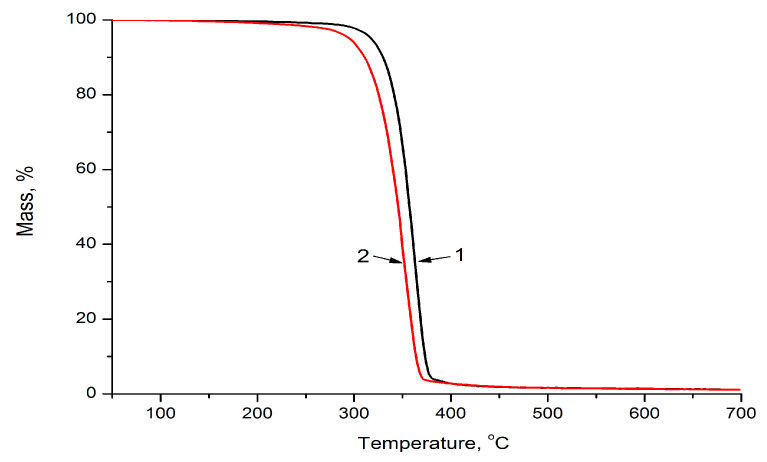
TGA thermograms of PLA/NR samples with 10% wt. of NR content: 1—initial, and 2—after 180 days of hydrolytic degradation.

Figure 5 shows typical mass loss curves, which are similar for all the fiber samples. The TGA data confirm the effect of water on PLA/NR fibers. After the experiment, the mass loss occurs at a lower temperature when compared to the initial sample. The maximum degradation temperature is shifted by 10–13 °C toward low temperatures for all the fiber samples.

UV radiation is another aggressive environmental factor. During photo-destructive processes, polymers and the products that are made from them change various properties and colors and lose their performance characteristics [32,33]. When studying the effect of UV radiation on polymer materials, irradiators with different wavelengths are used. The most active part of the UV spectrum does not reach the earth’s surface, but radiation in the range of 270–400 nm affects all creatures and objects, so researchers often use ultraviolet sources with wavelengths from the above range in model tests.

**Figure 6:**
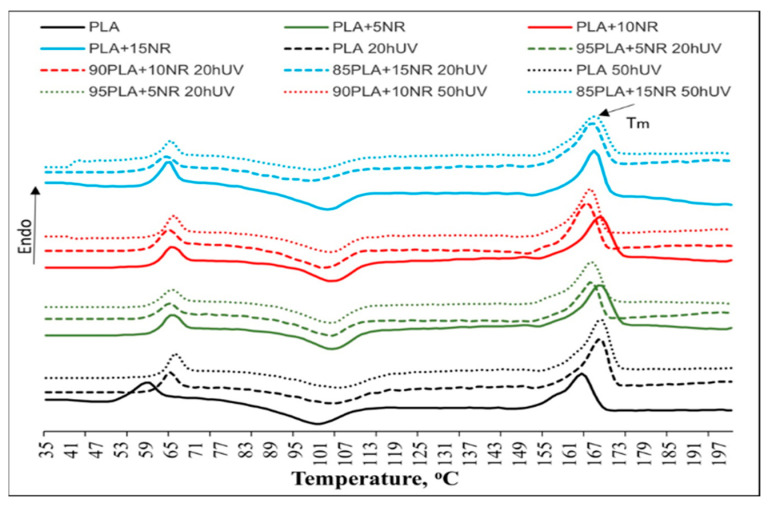
Thermograms of PLA/NR agrofiber samples from various compositions: initially and after UV radiation; λ = 365 nm for 20 and 50 h. The DSC method.

In this part of the study, it is important to determine the effect of NR on the photodegradation of nonwoven fiber samples, and the addition of NR increases or decreases the resistance of nonwoven fibers to UV radiation, which is very important for covering material used in open-ground conditions. The effect of UV irradiation on fiber was evaluated using the DSC method. Figure 6 shows the thermograms of the initial samples of PLA/NR fibers and after UV irradiation for 20 and 50 h. Table 3 shows the thermophysical characteristics of nonwoven fiber PLA/NR from different compositions after the influence of UV radiation.

From the DSC data, it follows that when exposed to UV radiation, the glass transition temperature (T_g_) of the PLA changes ambiguously. In pure PLA, the T_c_ increases by 7 °C, while the T_m_ increases by 6 °C and the degree of crystallinity increases by 7%. In samples of nonwoven fiber PLA/NR of the PLA, the T_g_ changes slightly, and the melting and crystallization temperature decreases by 2–3 °C.

The T_g_ of pure PLA fiber increases after 20 and 50 h of the experiment. This is an interesting fact. Usually, PLA photodegradation is accompanied by a decrease of the T_g_ value. It is possible that the effect of UV irradiation (λ = 365 nm) for 50 h causes an annealing effect, in which T_g_, T_m_, and the degree of crystallinity increase. The experiment will be continued. Perhaps after 150 or 250 h of photodegradation, the thermal characteristics of pure PLA fiber will start to decrease.

## 4. Discussion 

Interfacial interactions in the “polymer–polymer” systems are of great importance in matters of structure formation, in particular the effect of amorphous polymer-rubber on the structure and properties of crystallizing PLA. The thermophysical characteristics of initial PLA/NR nonwoven fiber were represented (Table 2). The results confirmed the immiscible behavior of the fiber composites, since almost no changes were observed in the glass transition temperature (T_g_) of PLA with the NR content. It was observed that the addition of NR with a content of 5–15% wt. increased the degree of PLA crystallinity by 2–4%. 

Adding rubber at an amount of 30–50 wt.% in the matrix of the crystallizing polymer [34] can significantly affect the structure and properties of the composite. In mixtures containing 30–50 wt.% of the second component, a phase inversion often occurs. In this case, the dielectric, mechanical, and rheological properties will be determined by the characteristics of the second component. Therefore, to improve elasticity and maintain the other characteristics of thermoplastics, the rubber additive should be in the range of 20–25%.

Regarding the influence of aggressive environments on polymers and polymer composites, such studies will be relevant if there are new promising polymer materials. The effect of water on polymers has been studied since the middle of the last century [35]. Water plays a crucial role in biological processes but can adversely affect the performance of polymers. It has been established that the hydrolysis of PLA begins with the diffusion of water molecules into amorphous regions. After that, the degradation continues in the boundary layers of the crystal domains [36]. The hydrolytic degradation of PLA has been studied quite extensively [37,38,39]. The current study shows that the adding of NR at a content of 5–15 wt.% into the PLA matrix practically does not affect the degree of water absorption of fibrous samples and does not prevent the degradation of nonwoven PLA/NR fibers, which is confirmed by the DSC and TGA data.

Ultraviolet irradiation is a powerful aggressive factor that causes the aging of polymers, as well as changes in their color, structure, and performance properties. The absorbing centers are most often carbonyl and other oxygen-containing groups. In the presence of oxygen, light initiates a chain oxidative reaction, radicals are formed, and then the process proceeds by a radical mechanism, like the process of thermal-oxidative degradation.

In [40], the photodegradation of PLA-based nanocomposites with the addition of polycaprolactone and montmorillonite (wavelength 254 nm) was studied. As a result of research [38], it was found that the PLA was actively exposed to ultraviolet light. After 16 h, the molecular weight decreased, and the polylactide was destroyed. The addition of montmorillonite delayed the photodegradation rate of the polylactide. The analysis of the accumulation of acetic anhydride by IR spectroscopy suggests that the pure polylactide is degraded faster than the investigated composites. The study of the photodegradation of film samples of pure PLA and its mixtures with PE under the same conditions showed a rapid disintegration of not only the amorphous but also the crystalline phase of PLA [25].

It should be noted that irradiation with a wavelength of 254 nm is a model medium. In this work, no rapid degradation of the PLA crystal phase was observed. A wavelength of 365 nm was used, which corresponded to a softer radiation, so under these conditions, the disintegration of the amorphous phase and an increase in the degree of PLA crystallinity in fibrous PLA/NR samples could be observed over 50 h of ultraviolet light. Further investigation of photodegradation under the above conditions will allow one to estimate the effect of irradiation under milder conditions and to compare the obtained data with those from irradiation with a wavelength of 254 nm.

## 5. Conclusions

Promising and biobased nonwoven PLA/NR fiber was obtained by electrospinning with an NR content of 5–15 wt.%. The addition of NR to the PLA matrix leads to a slight increase of the melting temperature and the degree of crystallinity.

It was determined that the addition of NR into the PLA matrix only increased the MFR value by 0.5 g/10 min at 15 wt.%. All samples of PLA/NR have a similar degree of water absorption. NR has no observable effect on the hydrolysis of PLA, which would be beneficial for the biodegradation of PLA modified by NR.

Regardless of the content of NR, all samples of PLA/NR fibers retain the ability for hydrolytic degradation and photodegradation, which is a very important aspect for potential use in agricultural applications such as covering materials or seed mats, which should be ecofriendly.

## Figures and Tables

**Table 1:** Chemical composition of natural rubber (NR), grade SVR 3 L.

Substance	Content, wt.%
Poly(cis-1,4-isoprene)	91–96
Protein and amino acids	2–3
Resins	2–3
Soot indicator	0.5
Volatiles	0.8
Transition metal compounds	<1
Other	<1

**Table 2:** Thermophysical characteristics of PLA/NR nonwoven fiber samples: initially and after 180 days of water degradation.

NR, wt.%	Initial Samples			After Water Influence for 180 Days		
	T_g_, °C (Δ ± 0.5 °C)	T_m_, °C (Δ ± 0.5 °C)	χ_cr_, % (Δ ± 1%)	T_g_, °C (Δ ± 0.5 °C)	T_m_, °C (Δ ± 0.5 °C)	χ_cr_, % (Δ ± 1%)
0	61	164	34	--	168	46
5	63	168	36	--	167	46
10	66	167	38	--	167	47
15	65	166	37	--	166	47

**Table 3:** Thermophysical characteristics of PLA/NR nonwoven fiber samples: before and after 20 and 50 h of UV-irradiation exposure (λ = 365 nm).

NR, wt.%	T_g_, °C (Δ ± 0.5 °C)			T_m_, °C (Δ ± 0.5 °C)			T_c_, °C (Δ ± 0.5 °C)			χ_cr_, % (Δ ± 1%)		
	0 h	20 h	50 h	0 h	20 h	50 h	0 h	20 h	50 h	0 h	20 h	50 h
0	60	66	67	164	168	169	101	104	106	33	38	40
5	65	65	66	168	167	166	105	104	103	36	38	38
10	66	63	65	169	163	167	105	102	104	37	39	39
15	65	65	66	168	165	167	104	103	101	36	37	38
